# Cardiopulmonary Protection of Modified Remote Ischemic Preconditioning in Mitral Valve Replacement Surgery: A Randomized Controlled Trial

**DOI:** 10.1155/2024/9889995

**Published:** 2024-06-24

**Authors:** Lianqin Zhang, Kang Zhou, Tianchu Gu, Jingjing Xu, Mengzhu Shi, Jiang Zhu, Jindong Liu

**Affiliations:** ^1^ Department of Anesthesiology The Second Affiliated Hospital of Soochow University, Soochow, Jiangsu 215008, China; ^2^ Jiangsu Province Key Laboratory of Anesthesiology Xuzhou Medical University, Xuzhou, Jiangsu 221004, China; ^3^ Department of Anesthesiology The Affiliated Hospital of Xuzhou Medical University, Xuzhou, Jiangsu 221004, China

**Keywords:** acute lung injury, cardiopulmonary function, mitral valve replacement, myocardial enzymes, remote ischemic preconditioning

## Abstract

**Background:** Remote ischemic preconditioning (RIPC) is reported to have early-phase and delayed-phase organ-protective effects. Previous studies have focused on the organ protection of a single RIPC protocol, and the clinical outcomes remain uncertain. Whether the modified RIPC (mRIPC) protocol performed repeatedly provides cardiopulmonary protection is still uncertain.

**Methods:** In this single-center, randomized, controlled trial, 86 patients undergoing elective mitral valve replacement (MVR) surgery were randomized 1:1 to receive either mRIPC or no ischemic preconditioning (control). Three cycles of 5 min ischemia and 5 min reperfusion induced by a blood pressure cuff served as the RIPC stimulus. mRIPC was induced at the following three time points: 24 h, 12 h, and 1 h before surgery. Blood samples were withdrawn at 10 min after intubation (T0), at 1 h after aortic declamping (T1), and at 6 h (T2), 12 h (T3), and 24 h (T4) after surgery to measure the serum concentrations of myocardial enzymes and other biomarkers, including cardiac troponin I (cTnI), which was the primary endpoint of this study. Creatine kinase isoenzyme (CK-MB), lactate dehydrogenase (LDH), inotropic score (IS), and inflammatory mediators were also measured. Blood gas analysis was conducted to calculate the PaO_2_/FiO_2_ ratio and A-aDO_2_, and the incidence of acute lung injury (ALI) was also recorded.

**Results:** mRIPC significantly decreased the serum concentrations of cTnI, CK-MB, and LDH at T2, T3, and T4 (*p* < 0.01), and the IS decreased compared with that in the control group (12.0 ± 1.0 vs. 14.2 ± 1.1, *p* < 0.01). In addition, the incidence of ALI in the mRIPC group was decreased (32.6% vs. 51.2%, *p* = 0.039), and the PaO_2_/FiO_2_ was higher at T4 (*p* < 0.05). Compared with those in the control group, the levels of interleukin-6 (IL-6) and tumor necrosis factor-*α* (TNF-*α*) were decreased at T1, T2, T3, and T4 (*p* < 0.05) in the mRIPC group, and the level of IL-10 increased at the same time.

**Conclusions:** mRIPC decreased the incidence of myocardial and lung injury in MVR surgery, providing new evidence for the clinical application of RIPC in valve surgery.

**Trial Registration:** ClinicalTrials.gov (NCT01406678).

## 1. Introduction

Cardiac heart disease is a major cause of death and disability worldwide [1]. Mitral valve injury caused by rheumatic heart disease seriously affects the health and quality of life of patients. At present, mitral valve replacement (MVR) under cardiopulmonary bypass (CPB) is still one of the major treatments for mitral valve disease. However, heart and lung ischemia–reperfusion (I/R) injury during CPB can increase the incidence of postoperative cardiopulmonary complications in patients [2].

Remote ischemic preconditioning (RIPC), induced by brief episodes of blood pressure cuff inflation and deflation on the upper arm or thigh, can help remote vital organs withstand a subsequent prolonged ischemic event [3]. RIPC has been proven to provide cardiopulmonary and renal protection in cardiac surgery [4, 5]. However, the results of two large multicenter studies in recent years demonstrated that RIPC had no significant effect on clinical outcomes, particularly in patients undergoing coronary artery bypass grafting (CABG) [6, 7]. The following protective effects of RIPC have hitherto been identified: the “early window of protection,” which occurs immediately after the stimulus, lasts 2–3 h [8], and the “second window of protection,” which is evident 12–24 h after the stimulus, could be maintained for up to 48–72 h [9]. The protection of the early phase is short but more effective in terms of increasing the tolerance of myocardial ischemia, while the delayed phase provides this protection more sustained and protects against myocardial stunning.

Most previous studies have focused on CABG surgery, while few studies have focused on cardiopulmonary function injury after valve surgery. Moreover, RIPC is mostly a single intervention, and the optimal stimulation mode (time, intensity) of RIPC is still controversial. In this study, a modified RIPC (mRIPC) method, which was performed at 24 h, 12 h, and 1 h before surgery, was used for intervention. We expect that the delayed-phase protection induced by performing mRIPC 24 h and 12 h before surgery and the two distinct windows of protection induced by performing mRIPC 1 h before surgery can overlap to enhance the cardioprotection. We conducted this single-center, randomized, controlled clinical trial to assess whether mRIPC could provide cardiopulmonary protection in patients undergoing MVR surgery.

## 2. Materials and Methods

### 2.1. Ethics and Registration

A single-center, prospective, randomized, clinical trial was conducted on patients undergoing elective MVR surgery. The study was approved by the ethics committee of the Affiliated Hospital of Xuzhou Medical University, Xuzhou, Jiangsu Province, China (XYFY2016-KL035-01). It was performed in compliance with the Declaration of Helsinki. All participants provided written informed consent.

### 2.2. Patients

Eligible patients were adults with mitral valve disease who were scheduled to undergo MVR surgery under CPB between November 2017 and August 2019. The exclusion criteria were as follows: urgent surgery, preoperative mechanical ventilator support, systemic infection, severe peripheral vascular disease, drug therapy with sulfonylureas and nicorandil, ejection fraction (EF) less than 40%, severe lung diseases such as chronic obstructive pulmonary disease (COPD), and peripheral arterial disease affecting the upper limbs or inclusion in other studies.

### 2.3. Randomization and Allocation

The patients were randomized in a 1:1 ratio to undergo either the mRIPC group or no preconditioning (control group). The randomization sequence was generated by a computer and kept in sealed envelopes. The allocation details were sealed in numbered and opaque envelopes, and each treatment allocation was revealed by an independent staff member opening the envelope before the first mRIPC treatment and was supervised by an independent statistician. Because of the setting of the control group, the patients and operators were informed of the intervention measures, but the statistician did not know the actual situation.

### 2.4. Intervention: mRIPC Protocol

**Figure 1:**
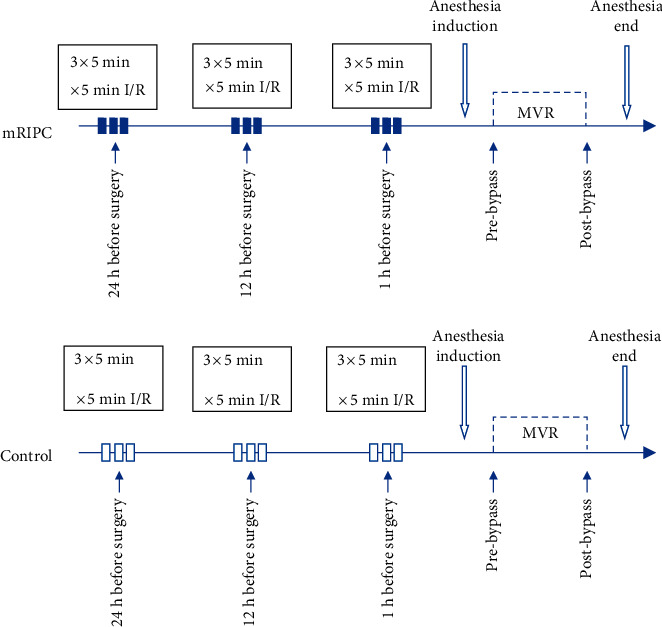
The mRIPC protocol of this study. I/R = ischemia–reperfusion, MVR = mitral valve replacement.

mRIPC was induced repeatedly at 24 h, 12 h, and 1 h before surgery to reinforce the protective effects of RIPC. The single RIPC protocol entailed three cycles of upper limb ischemia. A standard blood pressure cuff was placed on the right upper arm, and then, the cuff was inflated to 200 mm Hg for 5 min, followed by 5 min of cuff deflation [10]. The control group had no interventions. The details of the mRIPC protocol are provided in Figure 1.

### 2.5. Anesthetic and Surgical Management

Anesthesia was induced with intravenous etomidate (0.2 mg/kg), sufentanil (1 *μ*g/kg), and rocuronium bromide (0.6 mg/kg). All patients received continuous perioperative monitoring, including electrocardiography, pulse oximetry, radial arterial blood pressure (ABP), central venous pressure (CVP), rectal temperature, entropy index, and transesophageal echocardiography (TEE). After intubation, the patients were mechanically ventilated with a tidal volume of 8–10 mL/kg, an inspiratory to expiratory time ratio of 1:1.5, and a fractional inspired oxygen of 60%, and respiratory rates were adjusted to maintain arterial carbon dioxide between 35 and 45 mm Hg (Primus1 ventilator; Dräger Medical, Germany). Anesthesia was maintained with propofol (1.5 *μ*g/mL) by target-controlled infusion (TCI).

All patients underwent nonpulsatile CPB and received standard management. Heparin (3 mg/kg) was administered after sternotomy to achieve an active clotting time (ACT) longer than 480 s, and cardioplegic solution was used for cardioplegic arrest. During the CPB process, all patients used DelNido cardioplegia, which can maintain cardiac arrest for 90 min. The DelNido cardioplegia was reinfused almost every 90 min. The mean ABP was maintained at 60–70 mm Hg, and blood glucose levels were maintained at lower than < 200 mg/dL during CPB. Nitroglycerin (0.5 *μ*g/kg/min) was used after aorta declamping. After weaning from CPB, protamine was administered for the reversal of heparin, and the ratio of protamine to heparin was 1.2:1.5. All patients were transferred to the intensive care unit (ICU) and received standardized routine postoperative management.

### 2.6. Outcomes

The primary endpoint of this study was the concentrations of cardiac troponin I (cTnI). Secondary endpoints included creatine kinase isoenzyme (CK-MB) and lactate dehydrogenase (LDH) in serum, the inotropic score (IS), the autorebeat rate of the heart, and the incidence of reperfusion arrhythmia. The autorebeat was defined as the spontaneous recovery of the heartbeat without external stimulation after CPB, and reperfusion arrhythmias included the following types: serious bradycardia (HR < 40 beats/min) atrial fibrillation, frequent ventricular premature beats, and ventricular fibrillation. The IS was calculated with the following formula: (dopamine + dobutamine × 1) + (milrinone × 15) + (epinephrine + norepinephrine + isoproterenol × 100). The evaluation indexes of postoperative pulmonary function included the PaO_2_/FiO_2_ ratio and alveolar–arterial oxygen gradient (A-aDO_2_) according to the results of arterial blood gas analysis. The incidence of acute lung injury (ALI) was also assessed. ALI was defined as impaired oxygenation with a PaO_2_/FiO_2_ ratio of < 300 mm Hg, excluding bronchopneumonia, aspiration, cardiac origin pulmonary edema, and fluid overload. In addition, some inflammatory mediators, including interleukin-6 (IL-6), interleukin-10 (IL-10), and tumor necrosis factor-*α* (TNF-*α*), were also included. Lactic acid (Lac), as an indicator of metabolism, was also measured. All blood samples were collected at 10 min after intubation (T0), at 1 h after aortic declamping (T1), and at 6 h (T2), 12 h (T3), and 24 h (T4) after the completion of surgery. The ventilation time, ICU stay time, and length of stay (LOS) data were collected.

### 2.7. Blood Samples

The blood samples were collected and centrifuged immediately at 3000 rpm for 10 min at 4°C, and then, serum was frozen at −80°C for later analysis by enzyme-linked immunosorbent assay (ELISA) [11] with a commercially available kit (Wuhan Biofavor Biotech Services Co., Wuhan, China). Arterial blood samples were also collected for the blood gas analyses to calculate the PaO_2_/FiO_2_ ratio and A-aDO_2_ using a blood gas system (Roche Diagnostics GmbH, Germany).

### 2.8. Statistical Analysis

According to the results of the pilot study, the data with the minimum difference in cTnI concentration between the two groups at several time points after surgery were used to calculate the sample size. In this situation, the value in the mRIPC group was 3.11 ± 0.49, and the value in the control group was 3.50 ± 0.51. At least 36 patients were required per group when simulating the probability of a Type I error (*α*) at 0.05 and a Type II error at 0.1 (*β*). Considering a 15% dropout rate, the sample size was set at 43 patients per group.

Continuous data were analyzed with the Shapiro–Wilk test to determine whether they conformed to a normal distribution. Measurement data that obeyed a normal distribution are expressed as the mean ± standard deviation (mean ± SD), measurement data with a nonnormal distribution are expressed as the median (interquartile range (IQR)), and categorical variables are expressed as percentages. *T*-test was used to compare two groups of measurement data with normal distributions, the Mann–Whitney *U* test was used for measurement data with nonnormal distributions, and the chi-square test or Fisher's exact probability method was used for categorical data. Repeated measurement data at multiple time points were analyzed by repeated measurement analysis of variance (ANOVA). A *p* value less than 0.05 was considered significant. All statistical analyses were performed with SPSS statistical software, version 16.0 (SPSS Inc., Chicago, IL).

## 3. Results

**Figure 2:**
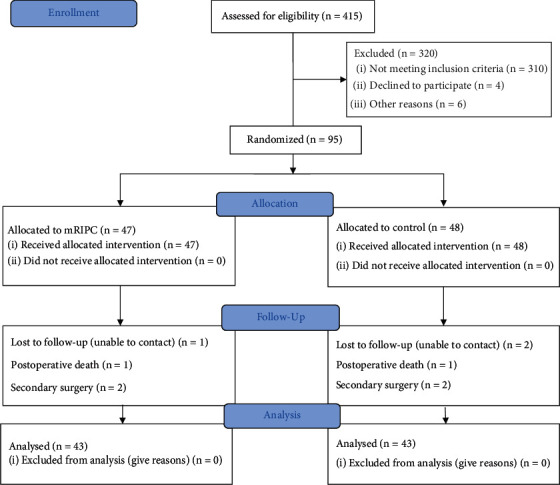
CONSORT flow diagram for the study.

In this study, 415 patients undergoing cardiac surgery were enrolled between November 2017 and August 2019, and 316 patients were excluded for the following reasons: 280 due to surgery types, 11 due to severe lung infection, 15 due to severe pulmonary hypertension, 6 due to EF < 30%, and 4 due to refusal to sign informed consent. Ninety-five patients were randomized to the mRIPC group (*n* = 47) or to the control group (*n* = 48). During the follow-up, some patients were further excluded due to perioperative death, secondary surgery, and the inability to contact patients. Forty-three patients in each group were included in the analysis (Figure 2).

The baseline demographic and clinical characteristics are shown in Table 1. The mRIPC group had a higher autorebeat rate of the heart (79.1% vs. 60.5%, *p* = 0.033) and a lower rate of reperfusion arrhythmia (16.3% vs. 32.6%, *p* = 0.044). In addition, there were no significant differences between the other baseline variables before and during surgery. The results of postoperative outcomes are presented in Table 2. The duration of postoperative mechanical ventilation was significantly decreased in the mRIPC group (18.6 ± 3.3 vs. 20.2 ± 3.8, *p* = 0.033).

**Figure 3:**
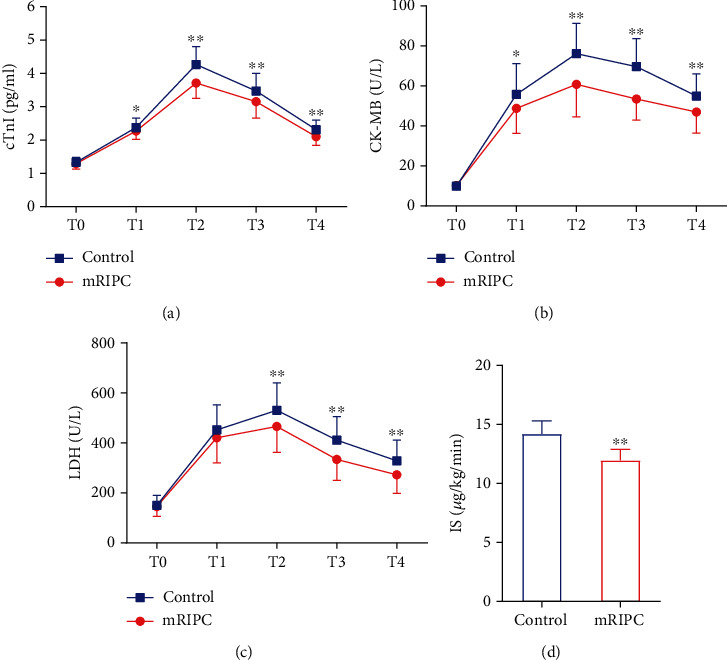
Serum myocardial enzyme content and IS in the two groups. *T*-test was used to compare two groups of measurement data, and repeated measurement data at multiple time points were analyzed by repeated measurement analysis of variance (ANOVA) in (a–c). *T*-test was used to compare two groups of measurement data in (d). (a) Serum cTnI content in the two groups. (b) Serum CK-MB content in the two groups. (c) Serum LDH content in the two groups. (d) IS in the two groups. ^∗^Compared with the control group, *p* < 0.05. ^∗∗^Compared with the control group, *p* < 0.01. CK-MB = creatine kinase isoenzyme; cTnI = cardiac troponin I; IS = inotropic score; LDH = lactate dehydrogenase; T0 = 10 min after intubation; T1 = 1 h after aortic declamping; T2 = 6 h after surgery; T3 = 12 h after surgery; T4 = 24 h after surgery.

There was no significant difference in cTnI, CK-MB, or LDH concentration between the two groups at T0. Compared to the control, mRIPC significantly decreased the serum concentrations of cTnI, CK-MB, and LDH at T2, T3, and T4 (*p* < 0.01), and the IS was also decreased (*p* < 0.01). In addition, the concentration of CK-MB at T1 was also lower than that in the control group (Figure 3).

**Figure 4:**
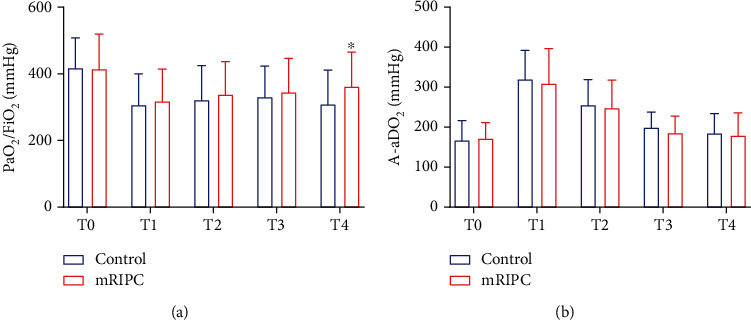
The postoperative outcome of pulmonary oxygenation function in the two groups. *T*-test was used to compare two groups of measurement data, and repeated measurement data at multiple time points were analyzed by repeated measurement analysis of variance (ANOVA). (a) PaO_2_/FiO_2_ in the two groups. (b) A-aDO_2_ in the two groups. ^∗^Compared with the control group, *p* < 0.05. T0 = 10 min after intubation, T1 = 1 h after aortic declamping, T2 = 6 h after surgery, T3 = 12 h after surgery, T4 = 24 h after surgery.

Compared to the control group, the PaO_2_/FiO_2_ of the intervention group was higher at T4 (362.0 ± 103.5 mm Hg vs. 309.4 ± 105.0 mm Hg, *p* = 0.022) (Figure 4(a)), and the incidence of ALI was significantly decreased (37.0% vs. 51.0%, *p* = 0.039) (Table 2).

**Figure 5:**
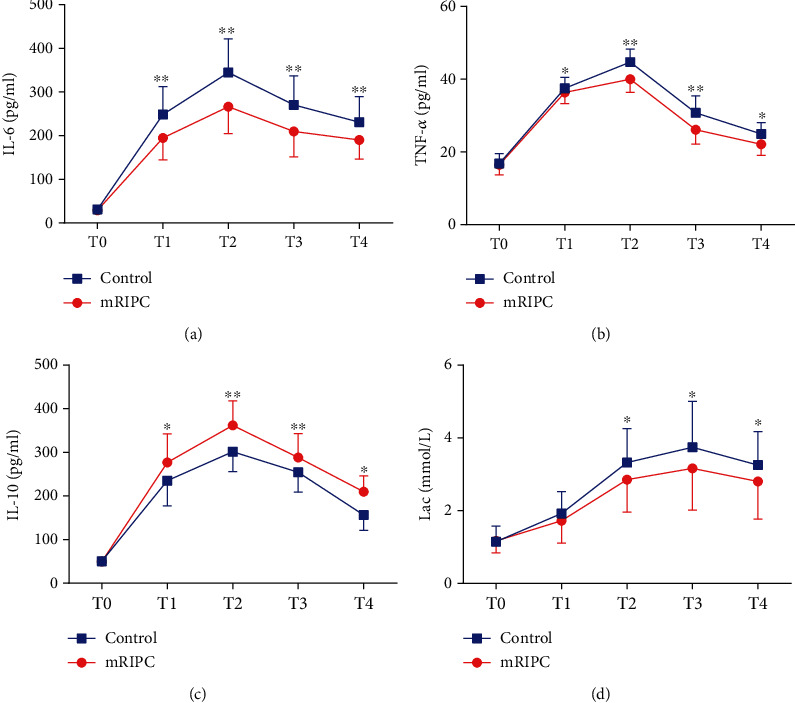
Comparison of inflammatory mediators and lactic acid values between the two groups at different time points after surgery. *T*-test was used to compare two groups of measurement data, and repeated measurement data at multiple time points were analyzed by repeated measurement analysis of variance (ANOVA). (a) IL-6 serum concentration in the two groups. (b) TNF-serum concentration in the two groups. (c) IL-10 serum concentration in the two groups. (d) Lac serum concentration in the two groups. ^∗^Compared with the control group, *p* < 0.05. ^∗∗^Compared with the control group, *p* < 0.01. IL = interleukin; Lac = lactic acid; T0 = 10 min after intubation; T1 = 1 h after aortic declamping; T2 = 6 h after surgery; T3 = 12 h after surgery; T4 = 24 h after surgery; TNF = tumor necrosis factor.

There were no significant differences in IL-6, IL-10, or TNF-*α* concentrations between the two groups at T0. At T1, T2, T3, and T4, mRIPC significantly decreased the level of IL-6 (*p* < 0.01) (Figure 5(a)). Compared with patients in the control group, patients in the mRIPC group had higher levels of IL-10 at T1 and T4 (*p* < 0.05) (Figure 5(c)), with the increase in IL-10 being more obvious at T3 and T4 (*p* < 0.01) (Figure 5(c)), and lower levels of TNF-*α* at T1 and T4 (*p* < 0.05) (Figure 5(b)), with the decrease in TNF-*α* being more obvious at T3 and T4 (*p* < 0.01) (Figure 5(b)). In addition, the difference in Lac between the two groups at T2, T3, and T4 was also statistically significant (*p* < 0.05) (Figure 5(d)).

## 4. Discussion

In this clinical study, we demonstrate for the first time that mRIPC induced three times can reduce the levels of myocardial injury–associated markers at multiple time points in patients undergoing MVR surgery, inhibit the inflammatory response, enhance oxygenation function, and decrease the incidence of ALI.

RIPC, as a noninvasive organ protection method initially proposed by Przyklenk et al. [12], has been shown to help remote organs withstand a subsequent prolonged ischemic event and provide multiorgan protection [13]. Previous preclinical studies [14, 15] and some clinical studies [16, 17] have shown that RIPC had a good protective effect on myocardial injury during cardiac surgery. The study results of Thielmann et al. [5] showed that RIPC provided perioperative myocardial protection and improved the prognosis of patients undergoing elective CABG surgery. However, some studies did not obtain positive results [18, 19]. These findings are in line with the report of the two large-scale prospective trials on RIPC in cardiac surgery [6, 7]. These contradictory results make the organ protection effects of RIPC controversial. Some researchers speculate that most of the existing studies were focused on patients undergoing CABG surgery; these patients often have transient myocardial ischemia before the operation, which may induce a protective effect similar to that of RIPC [20]. In this study, we selected patients who underwent elective MVR, and most of these patients had no history of myocardial ischemia before surgery. In addition, we also used a mRIPC method, which was carried out at three time points before surgery, so that the organ protection from the early and late time windows of RIPC can be overlapped. Our results demonstrated that mRIPC could significantly decrease the release of myocardial enzymes (cTnI, CK-MB, and LDH) at multiple time points (T2, T3, and T4) after surgery, and the IS was also decreased (*p* < 0.01). Myocardial enzymes represented by cTnI have been proven to be sensitive in the evaluation of myocardial injury, and the IS is also a good index to measure the use of vasoactive drugs after surgery [21]. In addition, mRIPC intervention has been confirmed to increase the autorebeat rate of the heart and reduce the incidence of reperfusion arrhythmia, which also shows its protective effect on the heart. Previous studies have shown that a single RIPC stimulation can reduce myocardial enzyme release and provide myocardial protection after valve surgery [22, 23], which is similar to our results. However, a recent study involving valve surgery and CABG did not support the protective effect of a single RIPC [24]. The combined use of propofol and volatile anesthetic regimens during surgery may be one of the reasons for this finding, and preoperative complications such as diabetes mellitus may also affect the results of the study. A previous experimental animal study has confirmed that the inherent protective effect of RIPC might be fully exploited by a volatile anesthetic itself [25]. In contrast, propofol has been considered by some studies to reverse the protective effect of RIPC [26, 27]. To observe the protective effect of RIPC more effectively, propofol was used to maintain anesthesia in this study.

To date, no study has addressed the optimal site and duration of the RIPC stimulus or the optimal number of repetitions. The study of Kim et al. [28] demonstrated that delayed RIPC (24–48 h before surgery) did not reduce myocardial enzyme release after CABG. It is worth mentioning that a study recently published by Meersch et al. [29] showed that high-dose RIPC could stimulate increases in tissue inhibitor of metalloproteinase-2 and insulin-like growth factor-binding protein 7 increases in patients refractory to low-dose RIPC and decrease the incidence of AKI after cardiac surgery. Both of them are methods to enhance the effect of RIPC. Our research provides a new direction for exploring optimal RIPC modalities in the future. Three complete preoperative interventions may cause pain or numbness, but our study showed that no patient had moderate or severe unbearable pain.

Our results showed that mRIPC was able to improve oxygenation and decrease the incidence of ALI after MVR surgery. The RCT study by Li et al. [30] reported that limb RIPC reduced the incidence of ALI by improving pulmonary oxygenation in patients without severe pulmonary disease after lung resection under propofol–remifentanil anesthesia. Another study found that limb RIPC may improve gas exchange after lobectomy by reducing the levels of EBC 8-isoprostane and other markers of oxidative lung injury [31]. Similar results were obtained from a recently published meta-analysis [32]. This meta-analysis included 10 clinical studies of cardiovascular surgery, and the final analysis showed that the RIPC group could reduce the expression of TNF-*α*, shorten the duration of ICU mechanical ventilation, and improve postoperative pulmonary function in patients undergoing cardiac surgery. Although the effect of RIPC on the incidence of major cardiovascular and cerebrovascular events after cardiac surgery is controversial, recent studies and our results show that RIPC has a positive protective effect on postoperative pulmonary function in patients undergoing cardiac surgery or noncardiac surgery. It is noted that although there was a statistical difference in oxygenation index at T4 after surgery, PaO_2_/FiO_2_ differences of 50 mm Hg in the 300 mm Hg range are not clinically important differences. However, the incidence of postoperative ALI was significantly decreased, and further research is needed to explore the protective effect of mRIPC on the lung after cardiac surgery.

Regarding the mechanism of RIPC organ protection, it is believed that ischemic stimulation can induce multiple pathways to play a protective role in target organs, which is achieved by regulating the nerve reflex and secretion of humoral factors [33]; adenosine, IL-6, TNF-*α*, IL-10, and other humoral factors have been confirmed to regulate the role of RIPC. Our study showed that mRIPC was able to reduce the expression of the proinflammatory cytokines IL-6 and TNF-*α* and increase the expression of the anti-inflammatory factor IL-10. In addition, compared with the control group, the serum Lac level was decreased in the mRIPC group, which was consistent with the findings of previous studies [34]. It is suggested that the ischemic inflammatory response induced by RIPC and the change in inflammatory mediators may be one of the vital mechanisms of RIPC in perioperative myocardial protection and lung protection, and more studies are needed to explore its mechanism in the future.

Our study has certain limitations. First, our study is not a double-blind design, which may affect the quality of the study. The main reason is that the research design did not include a sham RIPC group. Second, in this study, only the changes of some biochemical indexes such as myocardial enzymes were analyzed, but some long-term prognoses were not recorded in this study; the promotion of study conclusions may be limited. In addition, the RIPC stimulation mode in this study was three cycles, but some studies used four cycles, and the optimal stimulation mode of RIPC is still controversial. Last, this study only focused on the short-term clinical outcomes during the perioperative period, without longer term follow-up. The main outcome of myocardial protection was the change in myocardial enzymes, and less attention was paid to the major cardiovascular clinical events. In this study, there were no significant differences in ICU stay, LOS, or mortality during hospitalization between the two groups. We are preparing to design and implement a larger clinical study with a longer follow-up time to obtain more clinical data.

## 5. Conclusions

mRIPC can provide myocardial and lung protective effects in patients undergoing MVR surgery and can be performed safely and easily in clinical applications. Further studies are needed to investigate the protective effects of mRIPC on long-term clinical outcomes and other organs other than the heart and lung.

## Figures and Tables

**Table 1:** Baseline and clinical characteristics of the patients.

**Characteristic**	**mRIPC (** **n** = 43**)**	**Control (** **n** = 43**)**	**p** **value**
Age (years)	53.3 ± 8.8	54.7 ± 10.0	0.487
Sex (male/female)	18/25	20/23	0.157
Weight (kg)	62.6 ± 9.0	63.7 ± 11.7	0.662
LVEF (%)	60.8 ± 6.7	58.5 ± 8.2	0.171
FEV_1_/FVC (%)	81.5 ± 9.6	84.7 ± 9.2	0.334
Euro SCORE II (%)	3.1 ± 1.4	3.0 ± 1.5	0.714
Hypertension (*n*, %)	25 (58.1%)	23 (53.5%)	0.157
Diabetes mellitus (*n*, %)	12 (27.9%)	11 (25.6%)	0.187
Atrial fibrillation (*n*, %)	27 (62.8%)	30 (69.8%)	0.143
Crystalloid infusion (mL)	1229.0 ± 265.0	1302.0 ± 289.0	0.224
Colloid infusion (mL)	373.0 ± 139.0	387.0 ± 157.0	0.648
Urine (mL)	896.0 ± 253.0	845.0 ± 233.0	0.338
Type of procedure			
MVP (*n*, %)	14 (32.6%)	16 (37.2%)	0.162
MVP+TVP (*n*, %)	29 (67.4%)	27 (62.8%)	0.162
Valve type			
Mechanic (*n*, %)	42 (97.7%)	43 (100%)	0.500
Tissue (*n*, %)	1 (2.3%)	0 (0%)	0.500
Aortic cross-clamping time (min)	52.3 ± 7.5	54.9 ± 6.3	0.086
Bypass time (min)	75.1 ± 17.4	78.5 ± 23.2	0.450
Surgery time (h)	3.3 ± 0.6	3.4 ± 0.7	0.448
Autorebeat rate of the heart (*n*, %)	34 (79.1%)^∗^	26 (60.5%)	0.033
Reperfusion arrhythmia (*n*, %)	7 (16.3%)^∗^	14 (32.6%)	0.044

*Note:* The data are presented as the mean ± SD or number (%). *T*-test was used to compare two groups of measurement data with normal distributions, and the chi-square test or Fisher's exact probability method was used for categorical data.

Abbreviations: Euro SCORE, European System for Cardiac Operative Risk Evaluation; ICU, intensive care unit; LVEF, left ventricular ejection fraction; MVP, mitral valve replacement; TVP, tricuspid valvuloplasty.

^*^Compared with the control group, *p* < 0.05.

**Table 2:** Postoperative outcomes of the patients.

**Characteristic**	**mRIPC (** **n** = 43**)**	**Control (** **n** = 43**)**	**p** **value**
ICU ventilation time (h)	18.6 ± 3.3	20.2 ± 38	0.033
ICU stay (h)	39.0 ± 11.1	41.6 ± 8.4	0.23
Postoperative hospital stays (d)	13.1 ± 2.5	14.0 ± 4.2	0.201
Postoperative ALI (*n*, %)	16 (37.0%)^∗^	22 (51.0%)	0.039

*Note:* The data are presented as the mean ± SD or number (%). *T*-test was used to compare two groups of measurement data with normal distributions, and the chi-square test or Fisher's exact probability method was used for categorical data.

Abbreviation: ICU = intensive care unit.

^*^Compared with the control group, *p* < 0.05.

## Data Availability

No new data were generated or analyzed in support of this research.
